# The current landscape of predictive and prognostic biomarkers for immune checkpoint blockade in ovarian cancer

**DOI:** 10.3389/fimmu.2022.1045957

**Published:** 2022-10-27

**Authors:** Yufei Xu, Fengli Zuo, Huiling Wang, Jing Jing, Xiujing He

**Affiliations:** Laboratory of Integrative Medicine, Clinical Research Center for Breast, State Key Laboratory of Biotherapy, West China Hospital, Sichuan University and Collaborative Innovation Center, Chengdu, Sichuan, China

**Keywords:** ovarian cancer, immune checkpoint blockade, biomarker, immunotherapy response, prognosis

## Abstract

Immune checkpoint blockade (ICB) therapy has evoked a prominent shift in anticancer therapy. Durable clinical antitumor activity to ICB has been observed in patients with ovarian cancer (OC). However, only a subset of patients derive clinical benefit, and immune-related adverse events (irAEs) caused by ICB therapy can lead to permanent tissue damage and even fatal consequences. It is thus urgent to develop predictive biomarkers to optimize patient outcomes and minimize toxicity risk. Herein, we review current predictive and prognostic biomarkers for checkpoint immunotherapy in OC and highlight emerging biomarkers to guide treatment with ICB. The prevalent biomarkers, such as PD-L1 expression status, tumor-infiltrating lymphocytes, mutational burden, and immune gene signatures, are further discussed. We provide a state-of-the-art survey on prognostic and predictive biomarkers for checkpoint immunotherapy and offer valuable information for guiding precision immunotherapy

## Introduction

Immune checkpoint blockade therapies (ICBs) can circumvent tumor-mediated immune suppression and reinvigorate antitumor immune responses, in contrast with conventional therapeutic strategies that exert direct cytotoxicity against tumor cells (1, 2). Immune checkpoint inhibitors (ICIs) that target the programmed cell death protein-1 (PD-1)/programmed death receptor ligand-1 (PD-L1) axis or cytotoxic T lymphocyte antigen 4 (CTLA4) have achieved impressive success against various cancer types (3). ICIs have achieved remarkable clinical activity with durable disease control across multiple advanced tumors (4). Accordingly, several ICIs have been approved by the United States Food and Drug Administration (FDA) for patients with malignancies, including melanoma, lung cancer, triple-negative breast cancer (TNBC), colorectal cancer, gastric cancer, renal cell cancer, head and neck squamous cell cancer, bladder cancer, lymphoma and so on (5). Albeit substantial advancements in clinical therapy, only a minority of patients receiving ICIs derive benefits. In addition, ICB therapy is significantly restricted by the occurrence of immune-related adverse events (irAEs), resulting from immune hyperactivation and subsequent immune homeostasis disturbance. Severe adverse events can lead to permanent disorders and can be lethal in some cases (6). Therefore, there is intense interest in developing predictive and prognostic biomarkers for ICI therapy to better understand the benefits and risks driven by ICB and effectively select patients.

**Figure 1:**
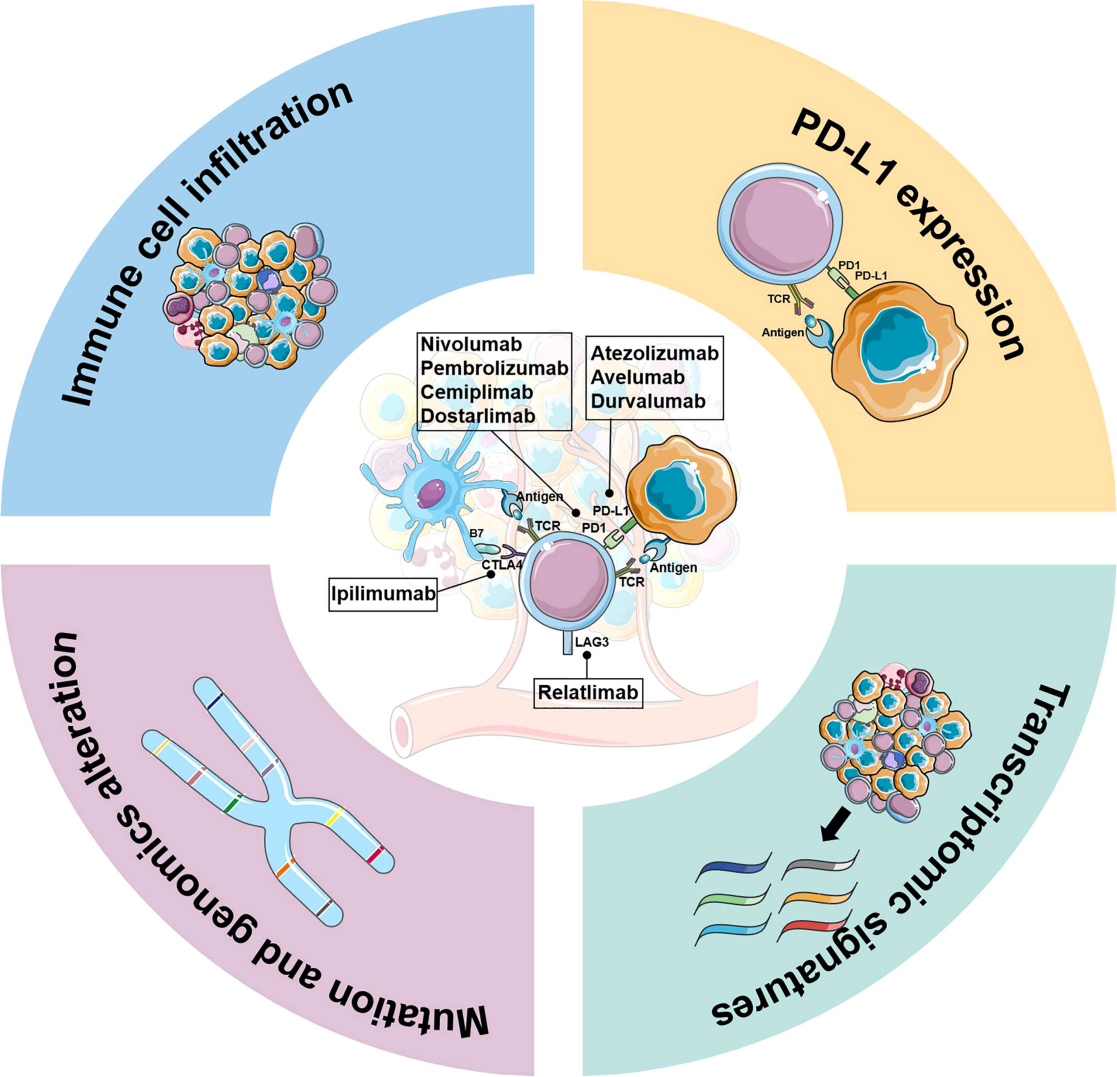
Biomarker development for immune-checkpoint inhibitor therapy in ovarian cancer. Key elements in biomarker development for immune checkpoint inhibitors therapy are briefly described, including PD-L1 expression, genomics alterations, immune cell infiltration, and transcriptomic signatures.

Manipulating the immune environment with ICIs is an attractive therapeutic approach for antitumor therapy in ovarian cancer (OC) (**Figure 1**). There has been considerable progress in utilizing ICB therapy for OC over the past few years (**Table 1**; **Supplementary Table S1**). However, there is still confusion regarding patient selection and the choice of therapeutic regimen for patients with OC, underscoring the need for effective biomarkers to predict response and remission. In this review, we attempt to summarize published original research and clinical trials involving biomarker assessment in OC receiving ICI therapy and discuss ongoing efforts to develop predictive biomarkers of responsiveness and outcomes.

**Table 1:** Predictive and prognostic biomarkers for checkpoint immunotherapy in ovarian cancer.

Categories	Biomarker	Association with favorable clinical outcome	Predictive versus prognostic	Tissue type for biomarker assessment	Possible assay type for biomarker assessment	Trial	Treatment	References
PD-L1	tumor PD-L1 expression	positive	predictive	tumor	IHC	NCT02674061	pembrolizumab	(7)
	tumor PD-L1 expression	positive	predictive	tumor	IHC	NCT02674061	pembrolizumab	(8)
	tumor PD-L1 expression	positive	predictive	tumor	IHC	NCT02674061	pembrolizumab	(9)
	tumor PD-L1 expression	positive	both	tumor	IHC	NCT02335918	varlilumab + nivolumab	(10, 11)
	PD-L1 expression both in tumor cells and immune cells	positive	both	tumor	IHC	NCT02580058	avelumab vs. avelumab + PLD vs. PLD	(12)
	tumor PD-L1 expression	potentially positive	predictive	tumor	–	NCT03558139	magrolimab + avelumab	(13)
	tumor PD-L1 expression	potentially positive	predictive	tumor	IHC	NCT02865811	pembrolizumab + PLD	(14)
	tumor PD-L1 expression	negative	predictive	tumor	IHC	NCT02873962	nivolumab + bevacizumab	(15)
TIICs	immune cell infiltration	positive	prognostic	tumor	RNA-seq	–	–	(16)
	CD8 expression	positive	both	tumor	IHC	NCT02580058	avelumab vs. avelumab + PLD vs. PLD	(12)
	immune score	positive	both	tumor	NanoString	NCT02657889	niraparib + pembrolizumab	(17)
	stromal tumor infiltrating mast cells (sTIMs)	negative	prognostic	tumor	IHC	–	–	(18)
Mutation and genomics alteration	the ratio of peripheral CD8 ^+^PD1^+^Ki67^+^ T cells to TMB	positive	prognostic	blood	DNA sequencing	NCT03029598	carboplatin + atezolizumab	(19)
	ARID1A loss/mutation	positive	predictive	tumor	DNA sequencing	–	–	(20)
	mutational signature 3	positive	both	tumor	DNA sequencing	NCT02657889	niraparib + pembrolizumab	(17)
	fraction of genome altered (FGA)	positive	both	tumor	DNA Sequencing	–	–	(21)
Transcriptomic signature	APOBEC3A expression	positive	both	tumor	qPCR	–	–	(22)
	immune-related genes	positive	prognostic	tumor	RNA-seq	–	–	(23)
	signal transducer and activator of transcription 1 (STAT1)	potentially positive	predictive	tumor	qPCR	–	–	(24)
	*CAPG* expression	negative	both	tumor	RNA-seq	–	–	(25)
	*LAYN* expression	negative	both	tumor	RNA-seq	–	–	(26)
	TGF-β score	negative	prognostic	tumor	RNA-seq	–	avelumab/nivolumab/pembrolizumab	(27)
	NAD^+^ metabolism-related genes (NMRGs)	negative	both	tumor	RNA-seq	–	–	(28)
	m6A-related gene signature	potentially negative	both	tumor	qPCR	–	–	(29)
	CXCL9	positive	prognostic	tumor	IHC	–	–	(30)
	CXCL11	positive	both	tumor	RNA-seq	–	–	(31)
	CXCL13	positive	both	tumor	IHC; IF	–	–	(32)
		potentially positive	both	tumor	RNA-seq	–	–	(33)
Peripheral blood biomarkers	increased IFNγ production	positive	predictive	blood	RNA-seq	NCT02484404	durvalumab + olaparib	(34)
	increased levels of CA-125	negative	predictive	blood	CA-125 test	–	–	(35)
	reduced levels of CA-125	potentially negative	predictive	blood	CA-125 test	NCT01772004	avelumab	(36)
	elevated VEGFR3 levels	negative	predictive	blood	RNA-seq	NCT02484404	durvalumab + olaparib	(34)
	ctDNA	negative	both	blood	bespoke ctDNA assays	NCT02644369	pembrolizumab	(37)

IHC, immunohistochemistry; IF, immunofluorescence; TIICs, tumor-infiltrating immune cells; PLD, pegylated liposomal doxorubicin; CA-125, cancer antigen-125; ctDNA, circulating tumor DNA; +, combination therapy; -, not available; /, or.

## PD-L1 expression

Direct measurement of PD-L1 expression is a logical biomarker for predicting response to anti-PD-1/PD-L1 therapies. PD-L1 immunohistochemistry (IHC) assay is now FDA-approved as a companion diagnostic biomarker to select patients most likely to benefit from ICI treatment for multiple cancer types, such as non-small cell lung cancer(NSCLC), metastatic TNBC, and melanoma (5).

The predictive value of PD-L1 expression was assessed in OC patients treated with anti-PD-1/PD-L1 antibodies (**Table 1**). KEYNOTE-100 (NCT02674061) investigated the clinical activity of pembrolizumab in patients with recurrent advanced OC and introduced PD-L1 stain score as a predictive biomarker, in which patients with higher PD-L1 expression (combined positive score≥10) had an increased overall response rate (ORR) and prolonged overall survival (OS) with pembrolizumab (8). More recently, Sanborn et al. evaluated the efficacy and safety of varlilumab plus nivolumab in patients with advanced solid tumors (10). Significantly, an absolute increase of 5% or more in tumor PD-L1 expression induced by treatment tended to improve progression-free survival (PFS) in OC (7.4 months *vs*. 3.5 months, p=0.07), whereas baseline pretreatment PD-L1 expression was not associated with ORR (10). Prespecified biomarker analysis in the JAVELIN-200 trial revealed a trend for prolonged PFS with the addition of avelumab to pegylated liposomal doxorubicin (PLD) compared with PLD alone among OC patients with PD-L1-positive tumors (12). Nevertheless, several trials yielded inconsistent or even contradictory results regarding the role of PD-L1 expression as a marker for predicting response to ICB and clinical outcomes in OC. Liu et al. (15) obtained the opposite results in evaluating the predictive and prognostic value of PD-L1 expression in recurrent OC patients receiving nivolumab and bevacizumab. Even patients with PD-L1-negative tumors (10/22) had higher therapeutic activity than those with PD-L1-positive expression (2/14) (15). In addition, several studies have shown that the expression of PD-L1 was not predictive of ICI outcome and prognosis in OC patients (36, 38–43). Potential reasons for these paradoxical results include the inability to accurately reflect PD-L1 status due to PD-L1 expression transiency and heterogeneity, differences in the disease status of patients, the poor uniformity between various detection assays, and the lack of standardized criteria and thresholds for assessing positivity (3, 44, 45). Therefore, PD-L1 status is likely insufficient to determine the suitability of ICI therapy for OC patients. Further refinement of the use of PD-L1 expression status as a robust biomarker for checkpoint immunotherapy is warranted.

## Tumor-infiltrating immune cells

TIICs can serve as an index to monitor the tumor microenvironment (TME) and play an increasingly important role in the immune response against cancer (46). Therefore, TIICs have also been speculated to be surrogate biomarkers for ICB immunotherapy in many types of cancer, including OC (**Table 1**). A comprehensive analysis of immune cells in patients with epithelial ovarian cancer (EOC) revealed a positive correlation between the infiltration of immune cells and the clinical outcome of EOC (16). The density of tumor-infiltrating lymphocytes (TILs), specifically CD8^+^ T cells, is a solid positive prognostic indicator for multiple cancer types regardless of ICI therapy. In fact, CD8 expression in tumors was predictive of clinical benefit with avelumab plus PLD treatment in OC (12). Of note, patients with dual PD-L1-positive and CD8-positive tumors seemed to benefit more from combination treatment than subgroups defined by only one of these biomarkers (12). Another potential predictor of ICI response is tumor-infiltrating mast cells (TIMs) within a tumor (**Table 1**). In high-grade plasmacytoid ovarian cancer (HGSOC), stromal TIMs (sTIMs) abundance was negatively associated with the ICB response (18). Remarkably, tumors with low sTIMs had enhanced effector functions of CD8^+^ T cells (18). This finding was corroborated in short-term HGSOC organoids. The effector molecules (GZMB and IFN-γ) on CD8^+^ T cells were marginally increased in organoids derived from low sTIMs tumors, compared to organoids from high sTIMs tumors (18). Overall, the abundance of sTIMs predicts a dismal prognosis in HGSOC patients treated with anti-PD-1 therapy.

Except for the spatial position and density of TIICs, their phenotype and activation status also impact the clinical benefit of ICIs (3). The immune-inflamed phenotype is usually accompanied by the expression of PD-L1 on infiltrating immune cells and tumor cells, which is associated with a better response to ICI therapy (3). In a trial investigating combination regimens with anti-PD-L1 antibody in women’s cancers, a trend toward a positive association of treatment response with the degree of PD-L1-positive TILs was observed (39). In contrast, melanoma patients with PD-L1-positive TILs had a significantly worse prognosis than those with PD-L1-negative TILs (P = 0.008) (47). Further investigations are needed to determine whether PD-L1-positive TILs are suitable to serve as predictors of ICB effectiveness. In addition, other non-neoplastic cells in the TME are also non-negligible, which are probably of biological significance. Therefore, increased awareness of the role of these distinct TME compartments is needed for comprehensive biomarker development to predict ICB response and prognosis.

## Mutation and genomics alterations

Tumor development and progression generally occur along with the acquisition and accumulation of mutations (45). Neoantigens generated by mutations may lead to T-cell infiltration, thereby better response to immunotherapy (48). In fact, several studies have attempted to evaluate somatic mutations as biomarkers for predicting ICB response in OC (**Table 1**). ARID1A mutation or loss was associated with immune microenvironmental factors in clear cell ovarian cancer (CCC), suggesting that ARID1A status has potential as a biomarker to guide decisions concerning patient selection for ICB therapy in CCC (20). The phase I/II trial (NCT02657889) reported two novel biomarkers for the combination of poly (adenosine diphosphate-ribose) polymerase (PARP) and PD-1 inhibitors in the treatment of platinum-resistant OC (17). Mutational signature 3 reflected homologous recombination deficiency (HRD) status, and positive immune score (IS) was a surrogate of interferon-primed exhausted CD8^+^ T cells in TME. Specifically, the presence of one or both of the above alternative markers was associated with significantly prolonged PFS (HR = 0.32), while concurrent absence showed no response to PARP/PD-1 inhibitors(ORR= 0%) (17).

Another metric, known as tumor mutation burden (TMB), is a strong predictor of ICB efficacy. Unfortunately, its predictive performance in OC is disappointing. No significant correlation was found between TMB and immunotherapy response in recurrent OC (21). Furthermore, BRCA1/2 mutations and HRD status also did not predict the clinical benefit of ICI in heavily pretreated patients with OC (21). Notably, additional exploratory analyses identified the fraction of genome altered (FGA) as a promising biomarker of response to ICI in OC, which can characterize global copy number alterations. High FGA was significantly associated with improved OS (HR = 0.49; log-rank P = 0.01) and PFS (HR = 0.54; log-rank P = 0.014) after ICI therapy in OC (21). The optimal cutoff for defining high *vs.* low FGA is unclear; therefore, the predictive capacity of FGA warrants further validation.

TMB was also explored in the phase I/II trial (NCT03029598), which evaluated pembrolizumab and carboplatin for recurrent or refractory ovarian, fallopian tube, or primary peritoneal cancer (19). Stratification by the ratio of peripheral CD8^+^PD1^+^Ki67^+^ T cells to tumor burden at baseline yielded a significant survival advantage. Patients with a low ratio (<0.0375) had a median OS of only 8.72 months, while those with a high ratio (≥0.0375) had a significantly longer median OS of 18.37 months (p=0.0099). However, no significant survival difference was observed when using CD8^+^PD1^+^Ki67^+^ T cell (p=0.53) or tumor burden alone (p=0.24) as stratification criteria (19). Overall, TMB alone does not clearly discriminate responders from non-responders in OC patients treated with ICIs.

## Transcriptomic signatures

Gene expression analysis can uncover global tumor and microenvironment features, providing promise for predicting the clinical benefit of checkpoint inhibitor strategies. Multiplex characterization of the TME and gene expression signatures have been proposed as effective methods to dissect the immune contexture and cancer cell-intrinsic features. According to TME information derived from transcriptome data of OC, Li et al. (23) established immune cell infiltration (ICI) scores and an immune-related gene prognostic model to predict the clinical benefits of OC patients undergoing immunotherapy. Signal transducer and activator of transcription 1 (STAT1) has been demonstrated to be associated with TME. A recent study found that STAT1 expression was positively correlated with PD-L1 expression and had the potential to predict the response to ICB in patients with EOC (24). Integrins are transmembrane receptors that mediate the connection between cells and their external environment (49–51).

Several immune-related gene signatures have been confirmed to predict the immunotherapeutic response in OC. The TGF-β regulated signaling pathway was noted to contribute to immunotherapy resistance in OC (27). A significant negative correlation between the TGF-β score and ICI-PFS was observed in OC, with an ICI-PFS of 16.6 months in the low TGF-β score group compared to 2.65 months in the high TGF-β score group (p = 0.0012). As the most common RNA modification, N6-methyladenosine (m6A) plays a key role in epigenetics (52). A risk model based on m6A-related targets has an excellent clinical prognostic stratification effect in advanced OC. Importantly, the high- and low-risk groups divided by this model have significant differences in TME contexture, suggesting that this model may be able to predict immunotherapy response in OC (29).

Chemokines have essential roles in modulating immune homeostasis and inflammatory responses (53). Accumulating findings suggest that chemokines can influence cancer cell proliferation, invasion, angiogenesis, and therapy resistance by recruiting immune cells and modulating the TME (54, 55). The prognostic and predictive values of the CXC chemokine family have been addressed in the setting of OC, including CXCL9, CXCL11, and CXCL13 (**Table 1**). Tumors with high CXCL9 expression had significantly prolonged OS, implying the feasibility of CXCL9 expression as a novel prognostic marker for high-grade serous ovarian cancer (HGSC) (30). Similarly, Fan et al. (33) found a significant positive correlation between the expression of CXCL13, FCRLA, PLA2G2D, and MS4A1 and a better prognosis of OC. Meanwhile, these potential therapeutic genes could reflect OC immune status and allow better predictions of who will respond to ICI. Furthermore, Yang etal. (32) examined the therapeutic effects of CXCL13 and PD-1 blockade in human HGSC tumors and mouse models. They found that CXCL13 can augment the efficacy of PD-1 checkpoint blockade in HGSC by shaping the antitumor microenvironment. CXCL13 can facilitate CXCR5^+^CD8^+^ T-cell recruitment to tertiary lymphoid structures. Furthermore, the combination of CXCL13, CD8, and CXCR5 was confirmed as a potential prognostic indicator or response biomarker for ICB therapy in patients with HGSC. CXCL11 expression has been demonstrated as a biomarker for predicting the response to anti-PD-1/PD-L1 therapy in a clinical trial of OC (31). In OC patients with HRD, tumors with high CXCL11 expression had a more robust immune response to PD-L1 blockade than those with low CXCL11 expression. Notably, the tumor-infiltrating immunophenotype and neoantigen burden were significantly elevated in CXCL11-high tumors.

In addition, several genes have been demonstrated to be associated with immunotherapy efficacy and prognosis in OC (**Table 1**). For example, Capping Actin Protein, Gelsolin-Like (*CAPG*) (25) and Layilin (*LAYN*) (26) appeared to be indicators of ICI outcome. Tumors with high *CAPG* or *LAYN* expression showed a significantly shorter survival time. In a study, the predictive significance of NAD^+^ metabolism-related genes (NMRGs) on immunotherapy response in patients with OC was examined. The high-risk score obtained by the NMRG-based model was also associated with a poorer prognosis (28). Apolipoprotein B mRNA editing enzyme catalytic subunit 3A (APOBEC3A) has been recognized as an indicator of genomic instability and may aid in predicting the prognosis and response to immunotherapy in OC (22).

## Peripheral blood biomarkers

In recent years, there has been great interest in developing blood-derived predictive biomarkers of ICI response, owing to its convenient and non-invasive sampling (56). Cancer antigen 125 (CA-125) is an important tumor biomarker specific to OC (57); thus, several studies have carried out exploratory research on the predictive role of CA-125 in OC patients treated with ICIs (**Table 1**). A phase II trial (NCT02608684), designed for evaluating the combination of pembrolizumab and chemotherapy in platinum-resistant OC, found CA-125 to be a reliable marker that reflected response and progression (42). In a retrospective study of EOC patients treated with ICI (35), the magnitude of increase in CA-125 levels within the first 12 weeks of treatment was significantly smaller in patients with clinical benefit than in those without benefit, suggesting a possible predictive role for the degree of CA-125 increase. In a phase 1b study of avelumab in patients with heavily pretreated OC, 12 patients with an objective response, of whom all 7 patients evaluable for CA-125 levels showed decreased CA-125 concentrations (36).

Dynamic monitoring of circulating tumor DNA (ctDNA) in plasma samples offers a meaningful direction for biomarker identification for immunotherapy in OC patients (37). A satisfying finding was that ctDNA concentration was related to clinical response and benefit, although the effect sizes were modest (37). Additionally, in a phase II trial of olaparib combined with durvalumab for OC, increased IFNγ production and elevated VEGFR3 levels in blood samples showed positive and negative correlations with PFS, respectively (p=0.023; p=0.017) (34).

## Conclusion and future directions

The clinical trials and original research outlined above have shown that classical biomarkers derived from the TME and tumor intrinsic features, such as PD-L1 expression, TMB, TIICs, and transcriptomic signatures, were correlated with ICI response and outcome in OC. Although these findings are intriguing, the implementation of these classical biomarkers has been hampered by inconsistencies and limitations. Promisingly, new biomarkers often designed as substitutes or complements to conventional biomarkers are constantly emerging, such as microbiome, tertiary lymphoid structures (TLSs), and tumor-associated antigens (TAAs). The potential of microbiome and its derived metabolome as biomarkers for predicting the efficacy of immunotherapy has been validated in melanoma (58), lung cancer (59), hepatobiliary cancer (60), and colorectal cancer (61). Several studies have demonstrated that clinical outcomes of immunotherapy for solid tumors are strongly correlated with the presence of TLSs, suggesting that TLSs may be a valid predictive indicator in the future (62). Elevated levels of carcinoembryonic antigen (CEA) have also been reported to negatively correlate with the prognosis of resected NSCLC patients receiving ICB therapy (63). More recently, a comprehensive predictive model for ICB response was developed across 16 different cancer types, which included the features of peripheral blood such as platelets, neutrophil-to-lymphocyte ratio, albumin, and hemoglobin (HGB) (64). These studies provide new perspectives to develop new biomarkers for OC patients treated with ICB therapy. The predictive values of these biomarkers in OC remain to be validated in routine clinical settings.

As evidenced by the fact that a single biomarker is often insufficient to determine the suitability of ICI therapy for OC patients, the combination of different biomarkers may be more valuable in predicting the clinical prognosis and therapeutic response to immunotherapy. Indeed, it has been proposed that the incorporation of dynamic and static biomarkers could improve decision-making to design tailored immunotherapy strategies. Moreover, the development of relevant biomarkers for the toxicity prediction of ICB therapy has become a research hotspot and is expected to offer effective ways to uncouple immunotherapy toxicity from its antitumor activity.

## Author contributions

YX and XH: conceptualization and writing-original draft preparation. FZ: visualization. YX, FZ, HW, JJ and XH: writing-review and editing. XH and JJ: supervision and funding acquisition. All authors have read and agree to the published version of the manuscript.

## Funding

This work was supported by (1) National Natural Science Foundation of China (No. 82172634 and 81902792); (2) Key Program of the Science and Technology Bureau of Sichuan (No. 2021YFSY0007); (3) 1.3.5 project for disciplines of excellence, West China Hospital, Sichuan University (No. ZYYC20013).

## Conflict of interest

The authors declare that the research was conducted in the absence of any commercial or financial relationships that could be construed as a potential conflict of interest.

## Publisher’s note

All claims expressed in this article are solely those of the authors and do not necessarily represent those of their affiliated organizations, or those of the publisher, the editors and the reviewers. Any product that may be evaluated in this article, or claim that may be made by its manufacturer, is not guaranteed or endorsed by the publisher.

## References

[1] Ott PA Hodi FS Robert C. CTLA-4 and PD-1/PD-L1 blockade: New immunotherapeutic modalities with durable clinical benefit in melanoma patients. Clin Cancer Res (2013) 19:5300–9. doi: 10.1158/1078-0432.CCR-13-0143 24089443

[2] Nishino M Ramaiya NH Hatabu H Hodi FS. Monitoring immune-checkpoint blockade: Response evaluation and biomarker development. Nat Rev Clin Oncol (2017) 14:655–68. doi: 10.1038/nrclinonc.2017.88 PMC5650537 28653677

[3] Havel JJ Chowell D Chan TA. The evolving landscape of biomarkers for checkpoint inhibitor immunotherapy. Nat Rev Cancer (2019) 19:133–50. doi: 10.1038/s41568-019-0116-x PMC6705396 30755690

[4] Gong J Chehrazi-Raffle A Reddi S Salgia R. Development of PD-1 and PD-L1 inhibitors as a form of cancer immunotherapy: A comprehensive review of registration trials and future considerations. J Immunother Cancer (2018) 6:1–18. doi: 10.1186/s40425-018-0316-z 29357948 PMC5778665

[5] Twomey JD Zhang B. Cancer immunotherapy update: FDA-approved checkpoint inhibitors and companion diagnostics. AAPS J (2021) 23:1–11. doi: 10.1208/s12248-021-00574-0 PMC7937597 33677681

[6] Postow MA Sidlow R Hellmann MD. Immune-related adverse events associated with immune checkpoint blockade. N Engl J Med (2018) 378:158–68. doi: 10.1056/NEJMra1703481 29320654

[7] Matulonis UA Shapira R Santin A Lisyanskaya AS Pignata S Vergote I. Antitumor activity and safety of pembrolizumab in patients with advanced recurrent ovarian cancer: results from the phase II KEYNOTE-100 study. Ann Oncol (2019) 30:1080–7. doi: 10.1093/annonc/mdz135 31046082

[8] Matulonis UA Shapira R Santin A Lisyanskaya AS Pignata S Vergote I. Final results from the KEYNOTE-100 trial of pembrolizumab in patients with advanced recurrent ovarian cancer. J Clin Oncol (2020) 38:6005–5. doi: 10.1200/JCO.2020.38.15_suppl.6005

[9] Nishio S Matsumoto K Takehara K Kawamura N Hasegawa K Takeshima N. Pembrolizumab monotherapy in Japanese patients with advanced ovarian cancer: Subgroup analysis from the KEYNOTE-100. Cancer Sci (2020) 111:1324–32. doi: 10.1111/cas.14340 PMC7156846 32012411

[10] Sanborn RE Pishvaian MJ Callahan MK Weise A Sikic BI Rahma O. Safety, tolerability and efficacy of agonist anti-CD27 antibody (varlilumab) administered in combination with anti-PD-1 (nivolumab) in advanced solid tumors. J Immunother Cancer (2022) 10:e005147. doi: 10.1136/jitc-2022-005147 35940825 PMC9364417

[11] Sanborn RE Pishvaian MJ Callahan MK Weise AM Sikic BI Rahma OE. Anti-CD27 agonist antibody varlilumab (varli) with nivolumab (nivo) for colorectal (CRC) and ovarian (OVA) cancer: Phase (Ph) 1/2 clinical trial results. J Clin Oncol (2018) 36:3001. doi: 10.1200/JCO.2018.36.15_suppl.3001

[12] Pujade-Lauraine E Fujiwara K Ledermann JA Oza AM Kristeleit R Ray-Coquard IL. Avelumab alone or in combination with chemotherapy versus chemotherapy alone in platinum-resistant or platinum-refractory ovarian cancer (JAVELIN ovarian 200): an open-label, three-arm, randomised, phase 3 study. Lancet Oncol (2021) 22:1034–46. doi: 10.1016/S1470-2045(21)00216-3 34143970

[13] Lakhani NJ Patnaik A Liao JB Moroney JW Miller DS Fleming GF. A phase Ib study of the anti-CD47 antibody magrolimab with the PD-L1 inhibitor avelumab (A) in solid tumor (ST) and ovarian cancer (OC) patients. J Clin Oncol (2020) 38:18–18. doi: 10.1200/JCO.2020.38.5_suppl.18

[14] Lee EK Xiong N Cheng SC Barry WT Penson RT Konstantinopoulos Lee Elizabeth PA K . Combined pembrolizumab and pegylated liposomal doxorubicin in platinum resistant ovarian cancer: a phase 2 clinical trial. Gynecol Oncol (2020) 159:72–8. doi: 10.1016/j.ygyno.2020.07.028 32771276

[15] Liu JF Herold C Gray KP Penson RT Horowitz N Konstantinopoulos PA. Assessment of combined nivolumab and bevacizumab in relapsed ovarian cancer: A phase 2 clinical trial. JAMA Oncol (2020) 5:1731–8. doi: 10.1001/jamaoncol.2019.3343 PMC6802049 31600397

[16] Liu S-Y Zhu R-H Wang Z-T Tan W Zhang L Wang Y-Q. Landscape of immune microenvironment in epithelial ovarian cancer and establishing risk model by machine learning. J Oncol (2020) 2021:1731–8. doi: 10.1155/2021/5523749 PMC8416376 34484333

[17] Färkkilä A Gulhan DC Casado J Jacobson CA Nguyen H Kochupurakkal B. Immunogenomic profiling determines responses to combined PARP and PD-1 inhibition in ovarian cancer. Nat Commun (2020) 11:1459. doi: 10.1038/s41467-020-15315-8 32193378 PMC7081234

[18] Cao K Zhang G Zhang X Yang M Wang Y He M. Stromal infiltrating mast cells identify immunoevasive subtype high-grade serous ovarian cancer with poor prognosis and inferior immunotherapeutic response. Oncoimmunology (2021) 10:1969075. doi: 10.1080/2162402X.2021.1969075 34527431 PMC8437532

[19] Liao JB Gwin WR Urban RR Hitchcock-Bernhardt KM Coveler AL Higgins DM. Pembrolizumab with low-dose carboplatin for recurrent platinum-resistant ovarian, fallopian tube, and primary peritoneal cancer: survival and immune correlates. J Immunother Cancer (2021) 9:e003122. doi: 10.1136/jitc-2021-003122 34531249 PMC8449961

[20] Kuroda Y Chiyoda T Kawaida M Nakamura K Aimono E Yoshimura T. ARID1A mutation/ARID1A loss is associated with a high immunogenic profile in clear cell ovarian cancer. Gynecol Oncol (2021) 162:679–85. doi: 10.1016/j.ygyno.2021.07.005 34272091

[21] Liu YL Selenica P Zhou Q Iasonos A Callahan M Feit NZ. BRCA mutations, homologous DNA repair deficiency, tumor mutational burden, and response to immune checkpoint inhibition in recurrent ovarian cancer. JCO Precis Oncol (2020) 4:665–79. doi: 10.1200/PO.20.00069 PMC7446408 32923884

[22] Xu F Liu T Zhou Z Zou C Xu S. Comprehensive analyses identify APOBEC3A as a genomic instability-associated immune prognostic biomarker in ovarian cancer. Front Immunol (2021) 12. doi: 10.3389/fimmu.2021.749369 PMC8568129 34745121

[23] Li X Liang W Zhao H Jin Z Shi G Xie W. Immune cell infiltration landscape of ovarian cancer to identify prognosis and immunotherapy-related genes to aid immunotherapy. Front Cell Dev Biol (2021) 9. doi: 10.3389/fcell.2021.749157 PMC8595115 34805159

[24] Liu F Liu J Zhang J Shi J Gui L Xu G. Expression of STAT1 is positively correlated with PD-L1 in human ovarian cancer. Cancer Biol Ther (2020) 21:963–71. doi: 10.1080/15384047.2020.1824479 PMC7583508 33043814

[25] Jiang S Yang Y Zhang Y Ye Q Song J Zheng M. Overexpression of CAPG is associated with poor prognosis and immunosuppressive cell infiltration in ovarian cancer. Dis Markers (2022) 2022:9719671. doi: 10.1155/2022/9719671 35186171 PMC8849939

[26] Li L Ma H Song C. LAYN acts as a prognostic biomarker in ovarian cancer by engaging T cell exclusion and dysfunction. Research Square (2022). doi: 10.21203/rs.3.rs-1943215/v1

[27] Ni Y Soliman A Joehlin-Price A Rose PG Vlad A Edwards RP. High TGF-β signature predicts immunotherapy resistance in gynecologic cancer patients treated with immune checkpoint inhibition. NPJ Precis Oncol (2021) 5:1–11. doi: 10.1038/s41698-021-00242-8 34921236 PMC8683510

[28] Lin L Chen L Xie Z Chen J Li L Lin A. Identification of NAD+ metabolism-derived gene signatures in ovarian cancer prognosis and immunotherapy. Front Genet (2022) 13. doi: 10.3389/fgene.2022.905238 PMC9243463 35783253

[29] Tan W Liu S Deng Z Dai F Yuan M Hu W. Gene signature of m6A-related targets to predict prognosis and immunotherapy response in ovarian cancer. J Cancer Res Clin Oncol (2022) 1–16. doi: 10.1007/s00432-022-04162-3 PMC11797572 36048273

[30] Seitz S Dreyer TF Stange C Steiger K Bräuer R Scheutz L. CXCL9 inhibits tumour growth and drives anti-PD-L1 therapy in ovarian cancer. Br J Cancer (2022) 126:1470–80. doi: 10.1038/s41416-022-01763-0 PMC9090786 35314795

[31] Shi Z Zhao Q Lv B Qu X Han X Wang H. Identification of biomarkers complementary to homologous recombination deficiency for improving the clinical outcome of ovarian serous cystadenocarcinoma. Clin Trans Med (2021) 11:e399. doi: 10.1002/ctm2.399 PMC8131501 34047476

[32] Yang M Lu J Zhang G Wang Y He M Xu Q. CXCL13 shapes immunoactive tumor microenvironment and enhances the efficacy of PD-1 checkpoint blockade in high-grade serous ovarian cancer. J Immunother Cancer (2021) 9:e001136. doi: 10.1136/jitc-2020-001136 33452206 PMC7813306

[33] Fan L Lei H Lin Y Zhou Z Shu G Yan Z. Identification of a gene set correlated with immune status in ovarian cancer by transcriptome-wide data mining. Front Mol Biosci (2021) 8. doi: 10.3389/fmolb.2021.670666 PMC8363306 34395521

[34] Lampert EJ Zimmer A Padget M Cimino-Mathews A Nair JR Liu Y. Combination of PARP inhibitor olaparib, and PD-L1 inhibitor durvalumab, in recurrent ovarian cancer: a proof-of-Concept phase II StudyPhase II study of olaparib with durvalumab in ovarian cancer. Clin Cancer Res (2020) 26:4268–79. doi: 10.1158/1078-0432.CCR-20-0056 PMC7442720 32398324

[35] Boland JL Zhou Q Iasonos AE O'cearbhaill RE Konner J Callahan M. Utility of serum CA-125 monitoring in patients with ovarian cancer undergoing immune checkpoint inhibitor therapy. Gynecol Oncol (2020) 158:303–8. doi: 10.1016/j.ygyno.2020.04.710 PMC7423717 32507515

[36] Disis ML Taylor MH Kelly K Beck JT Gordon M Moore KM. Efficacy and safety of avelumab for patients with recurrent or refractory ovarian cancer: Phase 1b results from the JAVELIN solid tumor trial. JAMA Oncol (2019) 5:393–401. doi: 10.1001/jamaoncol.2018.6258 30676622 PMC6439837

[37] Bratman SV Yang S Iafolla MA Liu Z Hansen AR Bedard PL. Personalized circulating tumor DNA analysis as a predictive biomarker in solid tumor patients treated with pembrolizumab. Nat Cancer (2020) 1:873–81. doi: 10.1038/s43018-020-0096-5 35121950

[38] Disis ML Patel MR Pant S Hamilton EP Lockhart AC Kelly K. Avelumab (MSB0010718C; anti-PD-L1) in patients with recurrent/refractory ovarian cancer from the JAVELIN solid tumor phase ib trial: Safety and clinical activity. Am Soc Clin Oncol (2016) 34:5533. doi: 10.1200/JCO.2016.34.15_suppl.5533

[39] Lee JM Cimino-Mathews A Peer CJ Zimmer A Lipkowitz S Annunziata CM. Safety and clinical activity of the programmed death-ligand 1 inhibitor durvalumab in combination with poly (ADP-ribose) polymerase inhibitor olaparib or vascular endothelial growth factor receptor 1-3 inhibitor cediranib in women's cancers: A dose-escalation, phase I study. J Clin Oncol (2017) 35:2193–202. doi: 10.1200/JCO.2016.72.1340 PMC5493052 28471727

[40] Zamarin D Burger RA Sill MW Powell DJ Jr. Lankes HA Feldman MD. Randomized phase II trial of nivolumab versus nivolumab and ipilimumab for recurrent or persistent ovarian cancer: An NRG oncology study. J Clin Oncol (2020) 38:1814–23. doi: 10.1200/JCO.19.02059 PMC7255977 32275468

[41] Mirza M Henriksen J Maenpaa J Christensen RD Waldstroem M Tandaric L. 1195 results of NSGO-OV-UMB1/ENGOT-OV30 study: A phase II study of durvalumab and oleclumab in patients with relapsed ovarian cancer (OC). BMJ Specialist Journals (2021) 31:A376. doi: 10.1136/ijgc-2021-ESGO.668

[42] Walsh CS Kamrava M Rogatko A Kim S Li A Cass I. Phase II trial of cisplatin, gemcitabine and pembrolizumab for platinum-resistant ovarian cancer. PloS One (2021) 16:e0252665. doi: 10.1371/journal.pone.0252665 34081738 PMC8174738

[43] Zsiros E Lynam S Attwood KM Wang C Chilakapati S Gomez EC. Efficacy and safety of pembrolizumab in combination with bevacizumab and oral metronomic cyclophosphamide in the treatment of recurrent ovarian cancer: A phase 2 nonrandomized clinical trial. JAMA Oncol (2021) 7:78–85. doi: 10.1001/jamaoncol.2020.5945 33211063 PMC7677872

[44] Gibney GT Weiner LM Atkins MB. Predictive biomarkers for checkpoint inhibitor-based immunotherapy. Lancet Oncol (2016) 17:e542–51. doi: 10.1016/S1470-2045(16)30406-5 PMC5702534 27924752

[45] Shen H Yang ES Conry M Fiveash J Contreras C Bonner JA. Predictive biomarkers for immune checkpoint blockade and opportunities for combination therapies. Genes Dis (2019) 6:232–46. doi: 10.1016/j.gendis.2019.06.006 PMC6997608 32042863

[46] Santoiemma PP Powell DJ Jr. Tumor infiltrating lymphocytes in ovarian cancer. Cancer Biol Ther (2015) 16:807–20. doi: 10.1080/15384047.2015.1040960 PMC4622931 25894333

[47] Ren M Dai B Kong YY Lv JJ Cai X. PD-L1 expression in tumour-infiltrating lymphocytes is a poor prognostic factor for primary acral melanoma patients. Histopathology (2018) 73:386–96. doi: 10.1111/his.13527 29637587

[48] Chic N Brasó-Maristany F Prat A. Biomarkers of immunotherapy response in breast cancer beyond PD-L1. Breast Cancer Res Treat (2021) 191:39–49. doi: 10.1007/s10549-021-06421-2 34676466

[49] Ganguly KK Pal S Moulik S Chatterjee A. Integrins and metastasis. Cell Adh Migr (2013) 7:251–61. doi: 10.4161/cam.23840 PMC3711990 23563505

[50] Bianconi D Unseld M Prager GW. Integrins in the spotlight of cancer. Int J Mol Sci (2016) 17:2037. doi: 10.3390/ijms17122037 PMC5187837 27929432

[51] Alday-Parejo B Stupp R Rüegg C. Are integrins still practicable targets for anticancer therapy? Cancers (Basel) (2019) 11:978. doi: 10.3390/cancers11070978 PMC6678560 31336983

[52] Xu T He B Sun H Xiong M Nie J Wang S. Novel insights into the interaction between N6-methyladenosine modification and circular RNA. Mol Therapy-Nucleic Acids (2022) 27:824–37. doi: 10.1016/j.omtn.2022.01.007 PMC8807973 35141044

[53] Tanegashima K Suzuki K Nakayama Y Tsuji K Shigenaga A Otaka A. CXCL14 is a natural inhibitor of the CXCL12–CXCR4 signaling axis. FEBS Lett (2013) 587:1731–5. doi: 10.1016/j.febslet.2013.04.046 23669361

[54] Milligan G Kostenis E. Heterotrimeric G-proteins: a short history. Br J Pharmacol (2006) 147:S46–55. doi: 10.1038/sj.bjp.0706405 PMC1760735 16402120

[55] Strieter RM Burdick MD Mestas J Gomperts B Keane MP Belperio JA. Cancer CXC chemokine networks and tumour angiogenesis. Eur J Cancer (2006) 42:768–78. doi: 10.1016/j.ejca.2006.01.006 16510280

[56] Di Capua D Bracken-Clarke D Ronan K Baird A-M Finn S. The liquid biopsy for lung cancer: state of the art, limitations and future developments. Cancers (2021) 13:3923. doi: 10.3390/cancers13163923 34439082 PMC8391249

[57] Razmi N Hasanzadeh M. Current advancement on diagnosis of ovarian cancer using biosensing of CA 125 biomarker: Analytical approaches. TrAC Trends Analytical Chem (2018) 108:1–12. doi: 10.1016/j.trac.2018.08.017

[58] Temraz S Nassar F Nasr R Charafeddine M Mukherji D Shamseddine A. Gut microbiome: a promising biomarker for immunotherapy in colorectal cancer. Int J Mol Sci (2019) 20:4155. doi: 10.3390/ijms20174155 PMC6747470 31450712

[59] Malczewski AB Navarro S Coward JI Ketheesan N. Microbiome-derived metabolome as a potential predictor of response to cancer immunotherapy. J Immunother Cancer (2020) 8:e001383. doi: 10.1136/jitc-2020-001383 33127655 PMC7604862

[60] Mao J Wang D Long J Yang X Lin J Song Y. Gut microbiome is associated with the clinical response to anti-PD-1 based immunotherapy in hepatobiliary cancers. J Immunother Cancer (2021) 9:e003334. doi: 10.1136/jitc-2021-003334 34873013 PMC8650503

[61] Shoji F Yamashita T Kinoshita F Takamori S Fujishita T Toyozawa R. Artificial intelligence-derived gut microbiome as a predictive biomarker for therapeutic response to immunotherapy in lung cancer: protocol for a multicentre, prospective, observational study. BMJ Open (2022) 12:e061674. doi: 10.1136/bmjopen-2022-061674 PMC9185567 35676015

[62] Trüb M Zippelius A. Tertiary lymphoid structures as a predictive biomarker of response to cancer immunotherapies. Front Immunol (2021) 12:674565. doi: 10.3389/fimmu.2021.674565 34054861 PMC8149953

[63] Clevers MR Kastelijn EA Peters BJ Kelder H Schramel FM. Evaluation of serum biomarker CEA and Ca-125 as immunotherapy response predictors in metastatic non-small cell lung cancer. Anticancer Res (2021) 41:869–76. doi: 10.21873/anticanres.14839 33517292

[64] Chowell D Yoo SK Valero C Pastore A Krishna C Lee M. Improved prediction of immune checkpoint blockade efficacy across multiple cancer types. Nat Biotechnol (2022) 40:499–506. doi: 10.1038/s41587-021-01070-8 34725502 PMC9363980

